# Bacteriological study of calf colisepticemia in Alage Dairy Farm, Southern Ethiopia

**DOI:** 10.1186/s13104-017-3038-2

**Published:** 2017-12-07

**Authors:** Mebrahtu Tedla, Kebede Degefa

**Affiliations:** Department of Veterinary Pharmacy and Biomedical Sciences, Faculty of Veterinary Medicine, The University of Gondar, P.O.box: 196, Gondar, Ethiopia

**Keywords:** Alage, Calf, Dairy farm, *E. coli*, Prevalence, Risk factors

## Abstract

**Objective:**

This study was designed to estimate the prevalence of *E. coli* which is the main cause of colisepticemia and the potential risk factors associated with the disease. A total of 74 calves less than 6 months age were selected for this study. For isolation and identification of *E. coli,* bacterial culture and biochemical tests were used.

**Result:**

Out of 74 calves selected for this study, 6 (8.11%) were positive for septicemic *E. coli*. Higher prevalence of 5 (8.93%) was recorded in Holstein Friesian breed than Boran breed 1 (5.56%). However, breed showed no significant difference on *E. coli* infections (P > 0.05). Higher prevalence of *E. coli* revealed below age of 30 days (17.39%) than calves aged between 30 and 90 days (8.33%) and above 90 days (0.00%). However, statistical association showed no difference (P > 0.05). Parity showed a significant difference in prevalence of *E. coli* (P < 0.05) in which infection increased with number of parity. Sex of the animal showed no association with infection of the calves (P > 0.05). Diarrheic calves showed higher prevalence (33.3%) than non-diarrheic calves (4.62%) with strong statistical association (P < 0.05). The present study showed a high prevalence of septicemic *E. coli* in the farm and intervention is strongly recommended.

## Introduction

Neonatal calf diarrhea (NCD) is the principal cause of calf mortality affecting the livestock industry globally [1, 2]. Septicemia caused by *E. coli* is a common disease of neonatal calves which causes a severe diarrhea and mortalities. The disease has a complex pathology which is known to have a multiple etiological agent [2, 3]. Some of the pathogenic organisms which causes calf diarrhea includes bacteria, viruses, and protozoa [4]. Co-infection make control of the disease more complicated [2]. Many reports showed that *E. coli* is one of the cause of enteropathogenesis in early ages of calves [5]. The incidence of calf diarrhea in neonates is reported in several studies and revealed a prevalence of 15–20% and rate of mortality showed from 1.5 to 8% [6]. In addition, the frequent presence of non-specific clinical signs ae the main factors influencing targeted treatments [7]. Diagnostic methods such as ELISA and PCR showed a better degree of detecting during mixed infections [8] and strain based diagnosis is important in providing appropriate information for proper therapeutic applications and finally control of the disease [2]. Various studies showed that different bacterial infections specifically the enterotoxigenic, enteropathogenic, enteroinvasive, and enterohemorrhagic *E. coli* leads to calf colisepticemia and causes neonatal mortalities particularly in non-vaccinated calves and those which deprived of colostrum [9–11]. The presence of large cattle population in developing countries [12] and the frequent and persistence of neonatal mortality and morbidity due to calf diarrhea without knowing the root cause of infection attract our attention to conduct this study. In these study population, we have noticed a clinical symptom of early diarrhea and mortalities which is a typical symptom of calf colisepticemia. As explained above, there are many pathogens causing the disease but due to the budget limitations of the study to characterize all, we focused only on characterizing and estimating the prevalence of *E. coli* and its associated risk factors in Alage Dairy Farm, Southern Ethiopia.

## Main text

### Methods

#### Study area

Alage Dairy Farm is located at Alage agricultural technical and vocational education training college (ATVET) situated at 217 km South West of Addis Ababa, Ethiopia in the vicinity of lakes Abijata and Shalla, located at 32 km to West of Bulbula. It belongs to the Agricultural Technical Vocational and Educational Training College under the Ministry of Agriculture and Rural Development [13].

#### Study population

Alage Dairy Farm was established in 1980 with a total of 300 cows and 4 bulls. These dairy animals have both the Holstein–Friesian and Boran breed origin and were purchased from Stella dairy farm located in Holeta and private farms located in capital of Addis Ababa. These animals are managed under intensive management systems. In this study, we focused on a total 74 new born calves aged below 6 months. These calves are owned by the college and used for teaching and commercial purposes.

#### Sampling strategies

This study was conducted in all calves found in the farm and the study was approved by the college animal ethics committee and we followed the ethical guidelines for care and use of animals.

#### Blood sample collection

Strict aseptic procedures were used [14] to collect blood samples from jugular vein of the calves [15]. A volume of 3 ml was collected into BD Vacutainer^®^ blood collection tubes and transferred immediately to laboratory for analysis.

#### Bacterial culture

A loop of blood sample was streaked on MacConkey agar plate incubated aerobically at 37 °C. After 24 h, the bacterial colonies were provisionally identified on the basis of morphology and color change due to lactose reaction to the media, medium size (1–3 mm diameter), bright pink to red with flat or elevated surface and white edges was observed, which is an indication of colony growth [16].

#### Biochemical characterization

For *E. coli* confirmation, the following biochemical characterization tests were performed. *Catalase test*: colonies were placed on clean slide and added 1–2 drops of 3% hydrogen peroxide, bubbles of oxygen were formed due to catalyze enzyme of the bacteria. *Motility test*: once a colony of bacterium was inoculated in semi-solid medium of sulphate indole motility media (SIM), the test organism shown by diffused turbidity in the medium after 24-h incubation at 37 °C. *Citrate utilization test.* This test was performed using simon citrate agar. *Methyl red test*: This test was performed to detect the production of sufficient acid during glucose fermentation. This was demonstrated through changing in color of methyl red indicator, which was added at the end of the incubation period and after the addition of five drops of indicator, there was the production of red color. *Triple sugar iron agar test:* Two reaction chambers within the medium tube was used: the slant (aerobic) and butt (anaerobic). Once this medium has been inoculated using straight wire on the slant and butt and incubated at 37 °C for 24 h, there was production of color change on the slant and butt. *Indole test:* This test was used to differentiate bacteria that are able to break down the amino acid tryptophan present in peptone water or in SIM agar to release indole. Indole was detected by kovac’s reagent or Ehrlich’s reagent. *E. coli* have characteristic of indole production. The major biochemical reactions used for identification of *E. coli* is summarized in Table 1.

**Table 1:** Growth characteristic and biochemical reaction of *E. coli*

Biochemical test	Characteristic of *E. coli*
Motility at 30 °C	Motile
Lactose fermentation	+
Indole production	+
Methyl red	+
Citrate utilization test	−
H_2_S production in TSI	−
Reaction in TSI	Yellow butt, yellow slunt
Catalase test	+

#### Data management and analysis

Data was analyzed using SPSS version 17 [17]. Prevalence of *E. coli* was calculated by dividing positive animals to a total number of animals studied. The explanatory variable age, breed, sex, parity of dam, and faecal consistency were considered as a potential risk factors and studied whether either has an association with septicemic *E. coli*. Fisher’s exact test method was used to interpret the statistical association. P-value less than 0.05 was required for significance.

### Result

#### Overall prevalence

A cross-sectional study to estimate the prevalence of septicemic *E. coli* in calves was conducted and blood samples were collected from calves of both indigenous (Boran) and exotic (Holstein Friesian) breeds under the age of 6 months. All the collected blood (N = 74) were subjected to primary and secondary biochemical tests and the overall prevalence of septicemic *E. coli* revealed 6 (8.11%). Different risk factors and their association with the prevalence of *E. coli* was also assessed and the result of each variable is showed as follows.

#### Age

calves in this study were categorized into three age groups, calves less than 30 days, aged between 30–90 days and above 90 days and a prevalence of 20.8, 4.5 and 0.00% was recorded in three of the age groups respectively. In this study, age was found to be significantly correlated with the prevalence of *E. coli* (P < 0.05). Association with prevalence of septicemic *E. coli* is given in Table 2.

**Table 2:** Prevalence of *E. coli* and associated risk factors

Risk factors	N	Positive	% Positive	95% CI	*P* value
Age					
< 30 days	24	5	20.8	(17.3–21.07)	
30–90 days	23	1	4.54	(3.45–6.13)	0.021
Total	27	0	0.00	(0.00)	
> 90 days	74	6	8.11	(5.31–9.32)	
Breed					
Holstein Frisian	56	5	8.93	(7.88–11.09)	
Borena	18	1	5.56	(6.14–8.54)	0.511
Total	74	6	8.11	(9.46–11.86)	
Parity of the dam					
One or two	19	5	26.3	(21.45–29.71)	
Three	30	1	3.33	(1.07–5.91)	0.023
Above three	25	0	0.00	(0.00)	
Total	74	6	8.11	(4.98–10.88)	
Sex					
Male	9	1	11.11	(12.31–15.31)	
Female	65	5	7.69	(7.99–11.03)	0.601
Total	74	6	8.11	(9.35–11.61)	
Faecal consistency					
Diarrheic	9	3	33.33	(30.11–36.13)	0.016
Non diarrheic	65	3	4.62	(2.93–5.32)	
Total	74	6	8.11	(7.33–10.89)	

#### Breed

In this study, two groups of breeds was considered; Boran and Holstein–Friesian with a prevalence of 8.93 and 5.56% respectively. However, the statistical analysis showed no significant association (P > 0.05) with the prevalence of *E. coli.* The prevalence and its association with the risk factors is described in Table 2.

#### Parity

This study targeted at three groups of dams (cow calved one or two times, cow calved three times and cow calved more than three) and the prevalence of *E. coli* showed 26.33, 3.33 and 0.00% in all the groups respectively. The present finding showed that parity has also a significant (P < 0.05) association with prevalence of septicemic *E. coli,* Table 2.

#### Sex

During the study, we expected that sex of the calve might have an implication on the infection rate or prevalence of *E. coli* due to the difference in the management system between male and female calves and it showed a prevalence of 11.11 and 7.99% in male and female calves respectively. However, sex failed to show significant difference (P > 0.05) with the prevalence of *E. coli* (Table 2).

#### Fecal consistency

The present study further indicated that the prevalence *E. coli* in diarrheic and non-diarrheic calves was 33.3 and 4.6% respectively and fecal consistency showed significant (P < 0.05) association with prevalence of *E. coli* as summarized in Table 2.

### Discussion

The result of the present study demonstrated the presence of septicemic *E. coli* in neonates in the farm. This study showed the overall prevalence of 8.11%. Similar study indicated the prevalence of septicemic *E. coli* in neonates as 51% [18]. However, this prevalence is higher because it targeted only clinically ill calves. Another study also showed a prevalence of 43.3% [19]. Such variation in the prevalence of the disease might be due to difference in sampling strategies. For example, random sampling method shows a clear picture of the epidemiology of the disease than purposive type of sampling strategies. A study by Ghanbarpour and Mohammad [20] revealed a prevalence of 22% and it is comparably higher than the prevalence of the present study. This is due to the age of the neonates which was less than 10 days and all were clinically ill with calf diarrhea. This is justifiable because neonates are more susceptible to infection comparing to the older calves.

#### Age

This study demonstrated the presence of statistical variation between different age group of calves. The prevalence between all age groups 5 (20.8%); below 30 days, 1(4.5%) between 30 and 90 days and 0 (0%) above 90 days was recorded. There was a significance (P < 0.05) association between different age groups. This age based difference in the prevalence of *E. coli* is supported by the rationale that younger calves are more susceptible to infection than older calves [9, 16, 21, 22]. Furthermore, *E. coli* can colonize the sterile intestine at early stage of neonate and those new born calves are more susceptible than adults specially if they take low colostrum [16].

#### Parity

Parity of the dam was considered to evaluate whether it contributes to the occurrence of septicemic *E. coli* and we showed a significance association (P < 0.05) between the dam parity and prevalence of *E. coli*. This finding agreed with Blood D.C.2002 [23] stated that colostrum of heifer is lower in immunoglobulin (Ig) content than older cows and colostrum deprived calves are highly susceptible for colisepticemia [3, 23].

#### Faecal consistency

This showed a significant (P < 0.05) association with *E. coli* infection. Calves having diarrhea were found to harbor septicemic *E. coli* 3 (33%) and showed higher prevalence than non-diarrheic calves found with prevalence of 3 (4.6%). This finding agreed with Radostits et al. [9] which stated diarrheic calves showed 31% of septicemic *E. coli*. In addition, calves with diarrhea are likely to have septicemic *E. coli* leading to pneumonia, shock and death [23, 24].

#### Breed

Even through there is no statistical significant (P > 0.05) association between breed and *E. coli* infection, a difference in prevalence is supported by Brooks and Morce [25] explained that different breed have different degree of susceptibility and exotic breeds are more susceptible than indigenous breeds [12].

#### Sex

In this study, there was no significant (P > 0.05) association between male and female individuals with septicemic *E. coli*. However, prevalence showed male have relatively higher prevalence (11.11%) than female (7.6%). This relative variation is supported by Thickett et al. [26] who explained female calf have a better attention and more managed because owners believed to replace those cows leaving the herd. Moreover, good management and provision of adequate nutrition lowers diseases susceptibility [10]. In conclusion, the present study clearly showed the presence of colisepticemia at herd level in the study area with the overall prevalence of 8.1%. This prevalence suggested that the disease in the farm has a possible risk of transmission from infected to healthy animals and it is a major threat for the production and productivity of the dairy cows and sufficient chemotherapeutic approach is recommended for the treatment of *E. coli* infection to control the disease epidemics.

## Limitations of the study

We found difficult to discover the epidemiology of the disease in the area due to small sample size and budget issues to discover all pathogens causing neonatal calf diarrhea. No prior work was done on the investigations of calf colisepticemia and this limited our scope of study and analysis of our result.
